# Bold-shy personality traits of globally invasive, native and hatchery-reared fish

**DOI:** 10.1098/rsos.231035

**Published:** 2023-08-23

**Authors:** Ellie Sales, Leia Rogers, Rafael Freire, Osmar Luiz, R. Keller Kopf

**Affiliations:** ^1^ School of Agricultural, Environmental and Veterinary Sciences, Charles Sturt University, Wagga Wagga, New South Wales 2650, Australia; ^2^ Gulbali Institute, Charles Sturt University, Albury, New South Wales 2640, Australia; ^3^ Research Institute for the Environment and Livelihoods, Charles Darwin University, Darwin, Northern Territory, Australia

**Keywords:** predator avoidance, animal personality, invasive alien species, behavioural syndrome, invasion syndrome, Australia

## Abstract

Bold behaviour of non-native species is hypothesized to facilitate invasion success, yet extreme boldness in wild and domesticated animals can be maladaptive. The purpose of this study was to compare individual behaviour among Australian native hatchery-reared (*n* = 33) and wild (*n* = 38) Murray cod (*Maccullochella peelii*) with invasive common carp (*Cyprinus carpio*; *n* = 30). Three laboratory tests measured individual behaviour: (1) emergence from a shelter, (2) exploration of a novel environment, and (3) approaching a predator. Wild invasive carp and hatchery-reared cod were generally faster and more likely to emerge and explore novel environments when compared with wild Murray cod. The ‘bold-type’ behaviours of hatchery-reared native cod were more like invasive carp than they were to ‘shy-type’ wild conspecifics, yet an important difference was that hatchery-reared cod spent substantially more time near a large predator while carp rapidly escaped. We suggest that these results are consistent with a bold-type invasion syndrome in invasive carp and learned boldness of hatchery-reared Murray cod. The propensity of invasive carp to rapidly explore and enter new environments, along with a fast predator escape response may have been important to their invasion success, while extreme risk-taking and predator naivety of hatchery-reared Murray cod may exacerbate post-release mortality rates in fisheries and conservation stocking programmes.

## 1. Introduction

Consistent individual differences in behaviour, often referred to as animal personality, is the subject of a large and rapidly growing body of literature demonstrating its influence on population dynamics, ecology, evolution, ecosystem functioning and conservation management [1,2]. Boldness is among the most widely studied personality traits and it is a measure of an individual's risk-taking reaction to a dangerous situation. Bold risk-taking individuals often have high rates of dispersal, exploration, aggression and fast life-history strategies which can form a ‘bold-type’ pace-of-life syndrome [3]. Conversely, a ‘shy-type’ pace-of-life syndrome has been associated with more limited dispersal, low aggression, low activity and slower life-history strategies within and across species and taxa.

Successful invasive species may exhibit bold-type invasion syndromes [4] whereby aggression, high activity, exploration and dispersal facilitate the rapid expansion of populations and boost competition with native species [5–7]. However, studies demonstrating bold-type invasion syndromes are relatively rare. In some cases, bold individuals are more common on the invasion front while shy individuals maintain and establish populations [8,9]. Although bold behaviour of non-native species is hypothesized to facilitate invasion success, there are a range of fitness costs associated with growth, food acquisition, reproduction and predation which appear to be context dependent and maintain a wide diversity of behavioural traits, from shy to bold, among individuals, populations and species [10].

Unlike boldness of successful invasive species, bold traits of hatchery-reared fish are generally considered maladaptive and detrimental to survival in the wild. Bold-shy differences have been documented between wild and domesticated [11,12] conspecifics of a wide variety of animal taxa. Learned behaviour in captivity can dampen behavioural responses to environmental and predator cues, usually facilitating more aggressive bold-type traits with important ecological consequences [13–15]. For example, high predation rates of hatchery-released fish in the wild have been attributed to bold behaviour and predator naivety [12,16,17]. Bold behaviour is well-established in hatchery-reared salmonids in the Northern Hemisphere, but relatively little research has been conducted on taxa from hatcheries outside of North America and Europe.

In the present study, we tested whether hatchery-reared Australian native fish were bolder than wild conspecifics and compared these with a globally widespread invasive species. Wild common carp (*Cyprinus carpio*), herein referred to as ‘carp’, were chosen for this study because they are a globally widespread invasive species [18–20], but have received relatively little attention to explore how behaviour [21–23] may have contributed to their invasion success. This invasive fish now makes up 70–90% of all fish biomass in the rivers and wetlands of southeastern Australia, and its abundance has been negatively correlated with native fish biomass and ecosystem changes [24–26]. Murray cod (*Maccullochella peelii*) are a territorial Australian native predator that has experienced historical population declines [27,28] and are the focus of hatchery release programmes and recreational fisheries [29,30]. We hypothesized that invasive and hatchery-reared fish would emerge and explore novel environments more frequently and quickly and would approach and spend more time near a predator in comparison with wild native fish. Wild native fish were hypothesized to emerge and explore novel environments less frequently and slowly and were expected to be reluctant to approach and spend time near the predator.

## 2. Material and methods

### 2.1. Subjects and housing

The subjects of this study consisted of hatchery (64 mm median total length; TL; electronic supplementary material, figure S1) and wild (116 mm median TL) sourced Murray cod, and wild common carp (*Cyprinus carpio;* 185 mm median TL). Fish ages included young of year (YOY) hatchery Murray cod (*n* = 19); one year old (1+) hatchery Murray cod (*n* = 14); YOY wild Murray cod (*n* = 17); 1+ wild Murray cod (*n* = 21), YOY wild carp (*n* = 19) and 1+ wild carp (*n* = 11). Fish age was determined at the end of experiments by removing and sectioning otoliths using traditional methods by experienced readers at Fish Ageing Services Pty Ltd. The hatchery fish were second generation from wild-caught breeding stock from the same southern Murray-Darling basin genetic population [31] and sourced from Uarah fisheries (Grong Grong, New South Wales). Therefore, differences in behaviour between wild and hatchery-reared Murray cod were assumed to be learned and not genetic. Wild Murray cod were collected by boat electrofishing and seining in canals of the Goulburn, and from the Murray river in Victoria, Australia. Carp were collected using electrofishing and fyke nets from the Goulburn and Murrumbidgee river in New South Wales, Australia. Field collections of fish were sampled as part of Victoria Fisheries permit no. RP1052 and New South Wales permit no. F93/158(C)-10.0.

Fish were transported in 50 l aerated containers and within 24 h of being collected were housed individually in aquaria. Fish were acclimatized in 35 l (length 290 mm × height 340 mm × width 440 mm) glass housing aquariums for 5 days before experiments commenced and were feeding prior to testing. Although fish could move freely, they were held individually in relatively small aquaria with black barriers which prevented social, physical and visual interactions which may have influenced test results. The temperature of the housing tanks was maintained between 21°C and 23°C with a pH of 7.2 and oxygen level of 8.3 mg l^−1^. Each housing tank had a constant outflow/inflow and filtration from biological filters. Automatic lights were set to a 12 : 12 cycle. The tops of the housing tanks were fitted with glass lids and steel mesh to prevent jumping. Each housing tank contained a 120 mm PVC pipe which provided shelter. Fish were fed a mixed diet of blood worms, chicken mince and fish fillets twice daily. Once the behavioural tests were completed, fish were euthanized in an ice slurry of 1 : 1 ice and water. Total length (mm) and mass (0.01 g) was recorded using a ruler and a digital scale.

### 2.2. Experimental design

**Figure 1.**
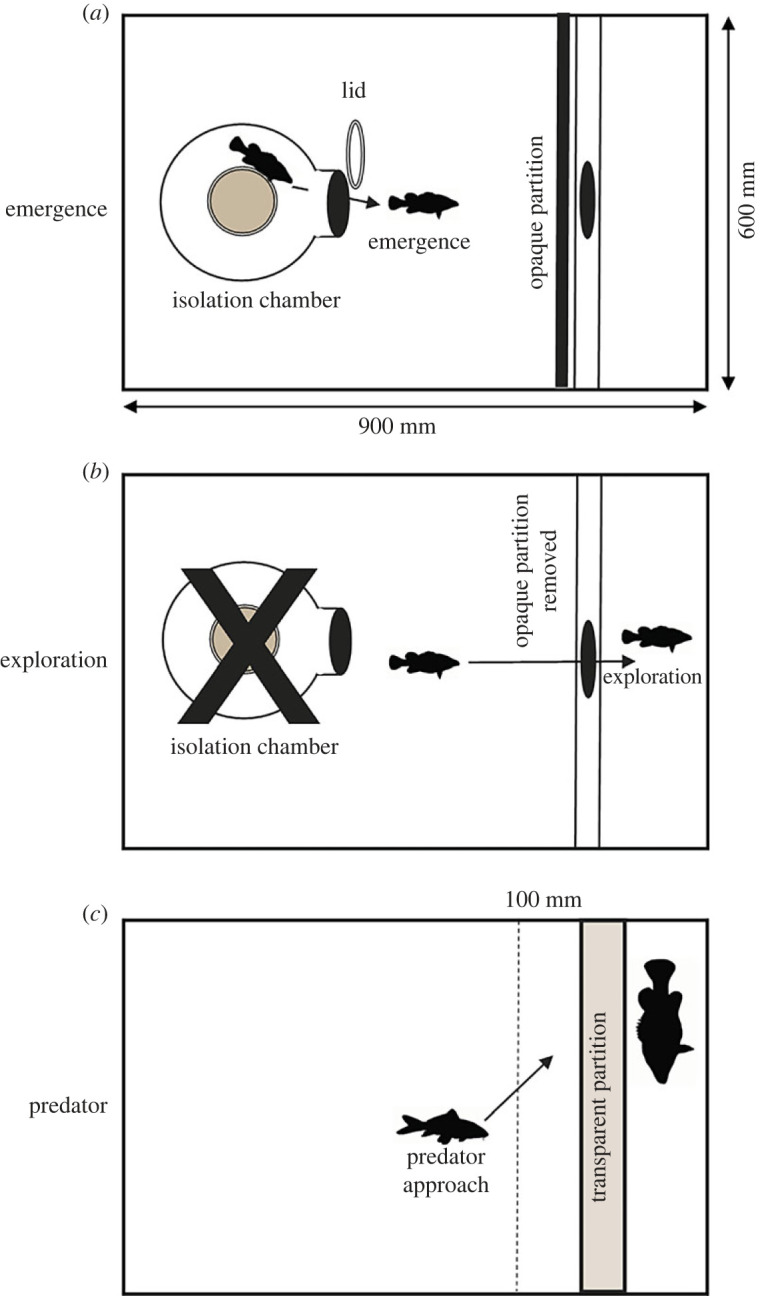
Diagrams illustrating the three behavioural tests including: emergence (*a*); exploration (*b*); and predator approach (*c*).

Three behavioural tests were conducted in 200 l testing tanks (length 900 mm × height 600 mm × width 600 mm) matched to the temperature and dissolved oxygen concentrations of housing aquaria. The testing tanks and partitions were arranged differently for each of the three tests (figure 1*a–c*). We used three methods to measure personality traits of individual fish: latency to emerge from shelter [32]; latency to explore a novel environment [33]; and willingness to approach a predator [34]. Bold-shy behaviour was assessed using three assays to test the consistency of personality traits across different contexts and over time [35].

#### 2.2.1. Test 1: emergence

An enclosed PVC isolation chamber (250 mm in diameter, 600 mm high) with a screw-cap lid was positioned in the 200 l testing tank as shown in figure 1*a*. Fish were transferred from housing tanks and placed into a novel isolation chamber. Each fish was given a 900 s acclimatization period, after which, the screw-cap was carefully removed from the isolation chamber. This experiment was completed when the entire body of the fish emerged, or if they did not emerge after 900 s. The time (s) the fish took to emerge was recorded using an overhead video camera (5231 Starlight, Dahua) to minimize disturbance. At the completion of Test 1, the isolation chamber was carefully removed from the testing tank and an opaque partition with a 150 mm hole was inserted (figure 1*b*), which marked the beginning of Test 2. Bolder fish were expected to emerge more often and faster than shy fish.

#### 2.2.2. Test 2: exploration

Test 2 commenced immediately following the completion (either 900 s or emergence from shelter) of Test 1. All individuals regardless of whether they successfully completed the emergence test or not undertook Test 2. The time taken for the entire subject to cross through the partition hole and enter the novel area (figure 1*b*), or not, was recorded. This experiment was completed when the subject entered the novel area, or after 900 s had passed without crossing. Following this test, fish were returned to the housing tanks for a minimum of 24 h before commencing Test 3. Bolder fish were expected to explore more often and explore more quickly than shy fish.

#### 2.2.3. Test 3: predator approach

The testing tank partition was assembled as shown in figure 1*c*. Fish were transported from housing tanks, placed in the 200 l testing tank and left to acclimatize for 600 s. After acclimatization, the opaque partition was lifted to reveal a larger predator (Murray cod; greater than 280 mm) separated from the testing fish by a clear partition. Whether or not (1; 0) the fish entered the zone less than 100 mm from the partition, nearest the predator was recorded and, if so, the duration of time the fish spent near the predator was recorded for up to 600 s. Bolder fish were expected to visit the predator more frequently and spend longer time periods near the predator.

### 2.3. Statistical analyses

We used generalized linear mixed-effects models (GLMMs; [36]) to test for differences in behaviour among each of the three tests: emergence; exploration; predator approach. Firstly, we fitted a GLMM using a binomial (logit) response (0 did not complete the test; 1 completed the test) using all individuals and all three tests. The model tested the probability of a fish completing a test, or not (0; 1) and evaluated differences in the fixed effects of the three tests (emergence; exploration; predator approach) and their interaction with fish group (wild Murray cod; hatchery Murray cod; wild carp) and length. Models were structured according to the formulae: binomial response (0;1)∼test (emergence; exploration; predator approach): fish group (wild Murray cod; hatchery Murray cod; Wild carp) with and without length as an interaction. Individual fish was modelled as a random effect (1/fish ID) in order to control for the non-independence individuals repeating each of the three tests. In fish that successfully completed a test (ie. emerged, explored or approached the predator) we compared times (s) by fitting GLMMs using a Poisson distribution, with fixed-effects, random effects and interaction terms the same as the binomial model. Using simple generalized linear models (GLMs), we also tested whether the fixed effects of emergence (binomial and time) and exploration (binomial and time) explained the probability of approaching a predator (0;1). Model structures were: predator approach (0;1)∼fish group:emergence (binomial and time as separate models). The same models were run separately for exploration. Emergence and exploration were correlated and therefore could not be used together as predictor variables in the same model. *Post hoc* pairwise comparisons were plotted using estimated log-odds ratios. Factors with variance inflation factor (VIF) scores of greater than 3.0 (e.g. emergence and exploration) were modelled separately. We determined the most parsimonious model using the Akaike information criterion (AIC), with a difference of 2 or more considered important. Analyses were performed in R programming language [37] using the packages ‘lme4’ [38] for GLMMs and ‘emmeans’ [39] for the *post hoc* tests.

## 3. Results

**Figure 2.**
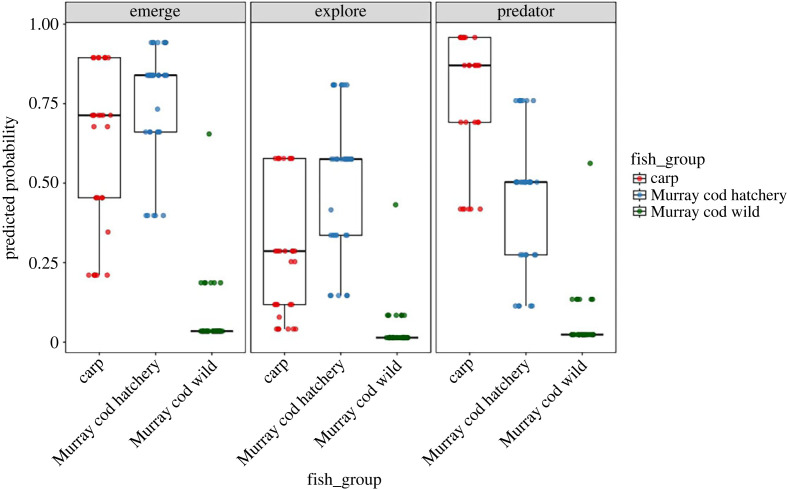
Boxplots of model predicted probability of completing (1) or not completing (0) bold-shy emergence, exploration and predator approach tests among invasive carp, hatchery and wild Murray cod.

Significant differences in behaviour were observed among fish groups, and individual behaviours were generally consistent among emergence, exploration, and predator approach probability tests (tables 1 and 2; figure 2). Wild invasive carp and hatchery Murray cod behaved similarly whereby individuals had a high probability of emerging, exploring a novel area and approaching a predator. By contrast, wild Murray cod rarely emerged, explored or approached the predator, and their modelled probability of completing each test was significantly lower than invasive carp and hatchery Murray cod in all tests. In the emergence test, 10% (4/40) of wild Murray cod emerged while 70% (23/33) and 61% (17/28) of hatchery Murray cod and wild carp, respectively, emerged. In the exploration test, 5% (2/40) of wild Murray cod explored the novel area, while 51% (17/33) and 32% (9/28) of hatchery Murray cod and carp, respectively, entered the novel area. In the predator approach test, 7% (3/38) of wild Murray cod entered the near-predator zone, while 45% (15/33) of hatchery Murray cod and 66% (18/27) of carp approached the near zone.

**Table 1.** Generalized linear mixed effects model results explaining the binomial probability of emergence, exploration and predator approaches of invasive carp, hatchery and wild Murray cod.

term	estimate	std. error	*z*-value	*p*-value
Emerge:carp	4.066	1.033	3.937	0.001
Explore:carp	2.241	0.971	2.308	0.05
Predator:carp	5.057	1.134	4.460	0.001
Emerge:hatchery Murray cod	4.940	1.053	4.690	0.001
Explore:hatchery Murray cod	3.593	0.978	3.673	0.001
Predator:hatchery Murray cod	3.301	0.970	3.402	0.001
Emerge:wild Murray cod	0.388	0.887	0.438	NS
Explore:wild Murray cod	−0.524	1.037	−0.505	NS
(Intercept) Predator:wild Murray cod	−3.516	0.827	−4.251	0.001

**Table 2.** Generalized linear mixed effects model results explaining differences in time (s) to emerge, explore and approach a predator in Murray cod and common carp that completed each test.

term	estimate	std. error	*z*-value	*p*-value
Emerge:carp	0.770	0.506	1.522	NS
Explore:carp	1.150	0.506	2.270	0.05
Predator:carp	−0.640	0.507	−1.263	NS
Emerge:hatchery Murray cod	0.686	0.492	1.394	NS
Explore:hatchery Murray cod	1.398	0.492	2.841	0.05
Predator:hatchery Murray cod	1.444	0.492	2.933	0.01
Emerge:wild Murray cod	1.369	0.100	13.687	0.001
Explore:wild Murray cod	0.460	0.114	4.033	0.001
(Intercept) Predator:wild Murray cod	4.070	0.442	9.206	0.001

**Figure 3.**
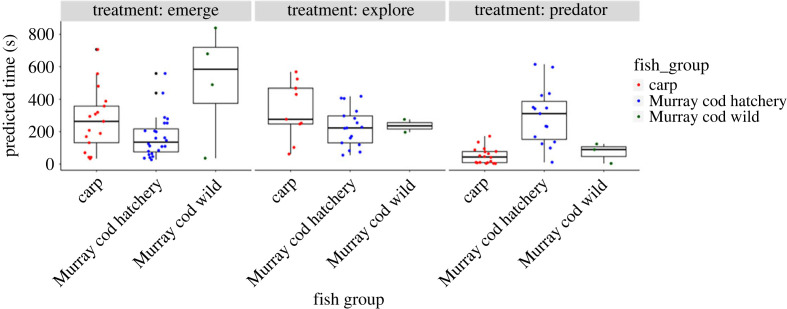
Boxplots of model predicted latency to emerge, explore and time (s) spent near a predator for invasive carp, hatchery and wild Murray cod only for individuals which completed each test (emerged; explored; or approached predator).

The only significant differences between invasive carp and hatchery Murray cod behaviour occurred in the predator approach test (figures 2 and 3). Invasive carp had the highest probability of approaching the predator followed by hatchery and wild Murray cod (figure 1; table 1). Invasive carp were highly likely to approach the predator, but spent substantially less time (modelled median = 44 s; figure 3, table 2) near the predator when compared with hatchery Murray cod (modelled median = 312 s) and wild Murray cod (modelled median = 90 s). Wild Murray cod were the slowest to emerge (figure 3), but were not different to other fish groups in time required to explore the novel environment.

**Figure 4.**
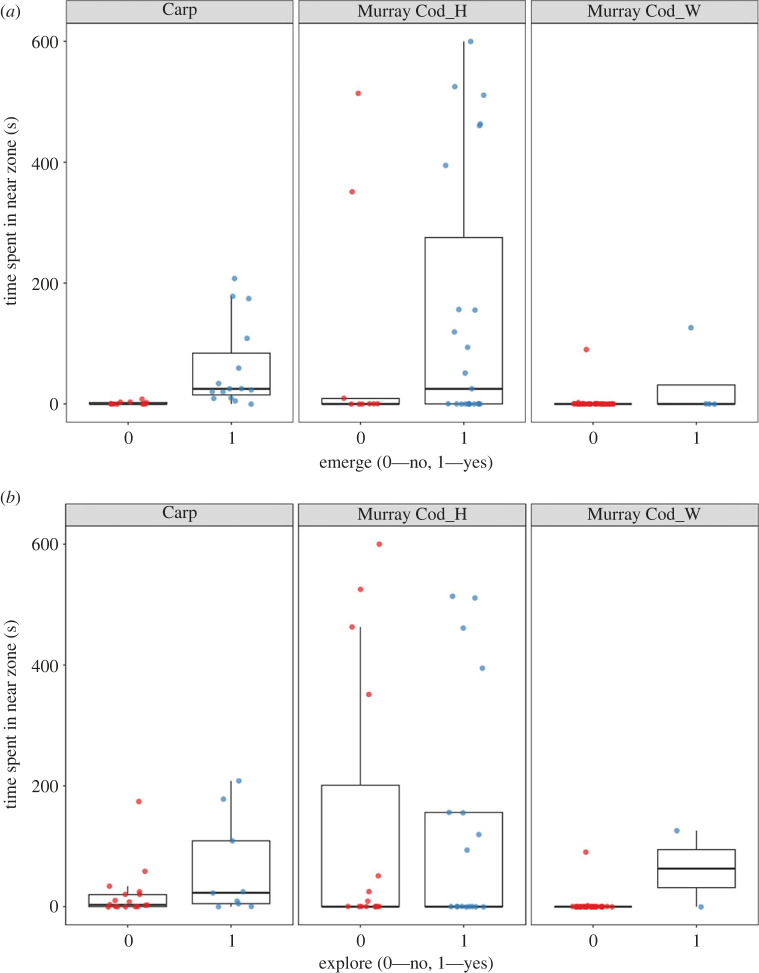
Boxplots showing model predicted time (s) spent in the near predator zone for invasive carp, hatchery and wild Murray cod in response to whether or not they completed emergence (top panel) and exploration (bottom panel) tests.

Individuals that emerged (GLM estimate 3.8 ± 0.25; z = 14.7; *p* < 0.001) and explored (GLM estimate 1.1 ± 0.07; z = 15.9; *p* < 0.001) generally spent longer time periods near the predator when compared with fish that did not emerge or explore (figure 4). The likelihood of individuals spending more time near the predator if they emerged or explored was consistent among most fish groups except for emergence of wild Murray cod and exploration of hatchery Murray cod. Emergence and exploration probabilities were positively correlated (greater than 0.60), and were therefore not used together as predictor variables. Length was not included in final GLMMs for probability of completing a test or time because the addition of length as an interaction term resulted in less parsimonious logistic probability (AIC = 306) and Poisson time (AIC = 1385) models when compared with respective models without a length interaction (AIC = 299; AIC = 1374). Despite length not being important (i.e. higher AIC values) across fish group and test interactions, there was a statistically significant negative effect of length on probability of Murray cod approaching the predator. This meant that larger Murray cod were less likely to approach the predator.

## 4. Discussion

Individual behaviour across emergence, exploration and predator approach tests provide evidence of ‘bold-type’ behaviours in globally invasive common carp. Bold behavioural traits are often correlated with fast life-history strategies which has been emphasized in the pace-of-life syndrome literature [3,11,40]. For instance, bold individuals often reproduce earlier, have high fecundity and fast growth rates [41]. Common carp are listed as one of the world's top 100 worst invasive alien species [19], and their adverse impacts on aquatic ecosystems and native fish are widely documented [24,26]. The success of common carp as a global invader has primarily been attributed to their fast-paced life-history strategy (e.g. high fecundity; early maturity; fast growth) and tolerance to a wide range of environmental conditions [18,20], but here we suggest that the bold-type behaviours of rapidly exploring and entering new environments—along with a fast predator escape response—may have contributed to their invasion success. By contrast to invasive carp, Murray cod are a longer-lived, slower growing and a less fecund species, broadly categorized as a K-selected or equilibrium life-history strategist [42]. Consistent with slow pace-of-life syndrome literature [3,40], wild Murray cod were considered to have ‘shy-type’ behavioural traits in comparison with hatchery conspecifics and wild carp. Our carp results were based on one invasive population in Australia and therefore tests of boldness in other parts of their global distribution and in other invasive species are warranted. Boldness in domesticated strains of common carp has previously been linked to angling vulnerability [23], and the species also appears to have high social learning and spatial memory skills allowing them to rapidly learn and locate food resources [21].

The personality traits of common carp are consistent with a growing body of literature [5–7,9] which support the hypothesis that successful invasive species may be bolder and have a higher propensity to disperse, enter and explore new environments when compared with some native species. Bold individuals of other species of carp have enhanced morphological defences when compared with shy individuals [22]. These studies and our results generally support the concept of an ‘invasion syndrome’ [4] as a potentially important dimension contributing to the success of introduced species. By contrast to our hypothesis, however, carp were the fastest to swim away from the predator. This result may have been reasonable to expect for a successful invasive fish, but conflicts with some previous studies [43] which have documented better predator avoidance responses of native prey in comparison with non-native prey when exposed to a native predator or its scent. Given that carp exhibited the fastest escape response, these data suggest that this invasive species is well-adapted to detect and avoid native predators in Australia despite only proliferating on the continent in the 1960s [44]. In our study, the native predator was a large wild Murray cod and the prey were juvenile conspecifics or wild invasive carp. Large Murray cod are a well-documented territorial cannibal and predator of carp in the wild and hatchery environment [28,42,45]. Although not tested directly here, the fast escape behaviours of wild invasive carp and wild Murray cod may be expected to reduce predation rates in comparison with hatchery-released fish in the wild. It is worth noting that larger Murray cod were less likely to approach the predator, and this could indicate that bigger fish perceived the predator as a territorial conspecific rather than a potential predator.

Unlike wild carp and wild Murray cod, long time periods (e.g. typically over 4–5 min longer) spent near the predator suggest that hatchery-reared cod were naive to predators. Hatchery-reared Murray cod were consistently bolder than wild conspecifics in all tests except for the time taken to explore the novel environment, and this discrepancy is probably explained by the small sample size (*n* = 2) of wild cod which completed the test. The substantial differences in boldness between hatchery and wild Murray cod, although expected, suggests that individual behaviour may be an important determinant of stocking success rates in Australia. An unexpected result from our study was that hatchery-reared Murray cod were not only bolder than wild conspecifics, but they were often as bold or bolder than invasive carp. Our hatchery fish results were consistent with literature documenting boldness in a range of fishes in captivity [13,23,46] and suggests that the hatchery practices in Australia are vulnerable to the same kinds of conditioning problems well-documented in salmonids and other groups of fish in the Northern Hemisphere. Given that hatchery fish in our study were spawned from wild brood stock sourced from the same southern genetic population [31] as wild juveniles in our study, genetics does not explain the behavioural differences we observed. Instead, we suggest that the bold-shy differences between wild and hatchery-reared Murray cod are learned behaviours that may be possible to mitigate using early life-history training [14,17,47], or by releasing fish prior to the development of learned behaviours.

Recovery programmes for Murray cod have used stock enhancement as a fisheries management tool for several decades [29], supplementing thousands of fish each year for recreational fishing and conservation purposes. Despite the widespread use of stocking, there has been relatively little assessment of its effectiveness [29], with most studies indicating that hatchery-reared fish have low survival and reproductive rates in the wild [12,30]. For juvenile fish, avoiding predation, using habitat and coping with varying abiotic conditions [48] are all likely to affect survival. Due to the widespread use of stocking programmes, further behavioural research aiming to improve predator avoidance, habitat use and reduce risk-taking before release from a hatchery is warranted. Previous research training hatchery Murray cod to respond to predators and use cover more effectively appears promising [47], and combined with our results should be seriously considered in fisheries hatchery programmes intended for supplementing wild fish stocks. Fish in our study were sourced from one hatchery, and therefore potential differences between hatcheries and life skills hatchery training methods [14,17] should be considered in future research.

## Data Availability

Raw data are available on Dryad: Personality traits and behaviour vary among invasive, native and hatchery-reared fish submitted with https://doi.org/10.5061/dryad.v41ns1s0h [49].
